# Editorial: Highlights in rehabilitation for musculoskeletal conditions 2021/22

**DOI:** 10.3389/fresc.2023.1130142

**Published:** 2023-01-26

**Authors:** Davide Piovesan, Alejandro González

**Affiliations:** ^1^ School of Engineering and Computing, Gannon University, Erie, PA, United States; ^2^ Tecnologico de Monterrey, Escuela de Ingeniería y Ciencias, Queretaro, Mexico

**Keywords:** rehabilitation, 3D printing, fear avoidance, musculoskeletal, spinal cord injury

**Editorial on the Research Topic** Highlights in rehabilitation for musculoskeletal conditions 2021/22

Rehabilitation for musculoskeletal conditions generally focuses on restoration of function and mobility. This can be achieved using a wide range of treatments, such as physical therapy, occupational therapy, and medication. The specific treatment plan will, of course, depend on the individual's needs and the specific condition being treated. The Research Topic “Highlights in Rehabilitation for Musculoskeletal Conditions 2021/22” aims to showcase recent contributions to the understanding of rehabilitation mechanisms. The Research Topic has received manuscripts concerning muscular rehabilitation in the context of a wide range of domains, such as fear avoidance, adolescent idiopathic scoliosis, Ehlers–Danlos syndromes (EDS) and generalized hypermobility spectrum disorders (GHSD), and incomplete spinal cord injuries. This editorial attempts to summarize the importance of the articles contributed.

Fear avoidance is a psychological phenomenon in which an individual avoids situations or activities due to fear of experiencing pain. It can lead to decreased physical activity and functional limitations, which can negatively impact the individual's ability to perform their duties. It can also contribute to the development of chronic pain and other musculoskeletal conditions. Treatment for this condition typically involves cognitive behavioral therapy, as well as physical rehabilitation to address any functional limitations. The authors of a brief research report, “*Development and validation of a military fear avoidance questionnaire*,” present the design of a tool for assessment of the Return to Duty Readiness Questionnaire (RDRQ) for use in soldiers with musculoskeletal pain Cooper et al. The RDRQ is based on the Fear Avoidance Components Scale (FACS) (1, 2), but it was necessary to adapt it to the military vocabulary before it could be successfully deployed among the target population. Although the study was interrupted by the COVID-19 pandemic, the initial outcomes are promising. The adapted RDRQ shows good correlation with the FACS, suggesting that the RDRQ can be used to measure fear avoidance in a population of active-duty members of the armed forces. The authors stated that evaluation of this instrument should resume shortly.

Adolescent idiopathic scoliosis (AIS) is a condition that causes the spine to curve abnormally. While its cause is unknown, AIS is most commonly seen in adolescents during the growth spurt before puberty. Treatment for AIS may include the use of braces to correct the curvature of the spine; in recent years, 3D printing technology has been used to create custom-fit braces. These braces are designed to be more comfortable and effective than traditional braces, and they can be adjusted as needed to accommodate the patient's changing body shape. However, the use of these 3D printed braces is still relatively new, and further research is needed to determine their long-term effectiveness. The article “*Immediate Outcomes and Benefits of 3D Printed Braces for the Treatment of Adolescent Idiopathic Scoliosis*,” contributed by Lue, Ng, and Hill Lou et al., presents the results of treating six participants using this approach.

Ehlers–Danlos syndromes (EDS) and other generalized hypermobility spectrum disorders (HSD) increase the risk of musculoskeletal injuries and chronic pain due to the presence of hypermobile joints. Exercise and rehabilitation can be an effective way to manage these conditions and improve overall function and quality of life. However, it is important for individuals with EDS and HSD to carefully select exercises and activities that are safe and appropriate for their condition. Furthermore, a model of care that includes exercise and rehabilitation therapy, education for self-management, and support in accessing relevant community resources can be an effective approach to the management of a variety of medical conditions (3, 4). By incorporating these elements into a comprehensive care model, health care providers can help individuals to achieve their goals and improve their overall health and well-being. Such a model of care is presented by Mittal et al. in their contributed article “*The GoodHope Exercise and Rehabilitation (GEAR) Program for People with Ehlers–Danlos Syndromes and Generalized Hypermobility Spectrum Disorders*”. This care model is notable due to its wide reach, and it can be applicable to various types of EDS.

Incomplete spinal cord injury (iSCI) denotes partial damage to the spinal cord. Individuals with iSCI may retain some function and sensation below the level of the injury but may still have difficulty with mobility. Fast walking can be especially challenging, as it requires rapid movements coordinated among body segments. The interaction between speed and lateral stability is an important factor in maintaining balance and preventing falls. In general, as an individual's speed increases, their lateral stability may decrease, making them susceptible to falling to the side. To evaluate the interaction between speed and lateral stability in gait, researchers typically use a combination of observational studies and laboratory tests. One such study, “*Stabilization Strategies for Fast Walking in Challenging Environments with Incomplete Spinal Cord Injury*,” is presented as part of this Research Topic by Cornwell et al. By testing two groups of age-matched individuals, the authors attempted to understand the effect of walking speed on lateral stability. They found that participants who had suffered spinal cord injuries unconsciously prioritize stability over speed. In their experiment, participants were required to walk on a treadmill following two path patterns (along a straight line vs. performing a lane change) under unperturbed or perturbed conditions. Perturbation was induced by tugging at the participant's waist. Their results showed that walking speed had no effect on stability in the unperturbed condition, and that participants with iSCI required more time to complete the lane change maneuver. According to the authors, this suggests the use of a different walking strategy in comparison to participants with no iSCI.

The articles presented in this Research Topic cover a wide range of subjects, but there is a collective focus on the implementation of novel techniques that could improve the results of musculoskeletal rehabilitation. These studies recognize the need for further work to better understand the effects of these techniques among their corresponding populations.
